# Hypoxia potentiates monocyte-derived dendritic cells for release of tumor necrosis factor α via MAP3K8

**DOI:** 10.1042/BSR20182019

**Published:** 2018-12-14

**Authors:** Laurent M. Paardekooper, Maura B. Bendix, Andrea Ottria, Lieke W. de Haer, Martin ter Beest, Timothy R.D.J. Radstake, Wioleta Marut, Geert van den Bogaart

**Affiliations:** 1 Department of Tumor Immunology, Radboud Institute for Molecular Life Sciences, Radboud University Medical Center, Nijmegen, The Netherlands; 2 Department of Rheumatology and Clinical Immunology, University Medical Center Utrecht, Utrecht; 3 Laboratory of Translational Immunology, Department of Immunology, University Medical Center Utrecht, Utrecht; 4 Department of Molecular Immunology, Groningen Biomolecular Sciences and Biotechnology Institute, University of Groningen, Groningen, The Netherlands

**Keywords:** dendritic cells, hypoxia, inflammation, mitogen-activated protein kinases, tumour necrosis factors

## Abstract

Dendritic cells (DCs) constantly sample peripheral tissues for antigens, which are subsequently ingested to derive peptides for presentation to T cells in lymph nodes. To do so, DCs have to traverse many different tissues with varying oxygen tensions. Additionally, DCs are often exposed to low oxygen tensions in tumors, where vascularization is lacking, as well as in inflammatory foci, where oxygen is rapidly consumed by inflammatory cells during the respiratory burst. DCs respond to oxygen levels to tailor immune responses to such low-oxygen environments. In the present study, we identified a mechanism of hypoxia-mediated potentiation of release of tumor necrosis factor α (TNF-α), a pro-inflammatory cytokine with important roles in both anti-cancer immunity and autoimmune disease. We show in human monocyte-derived DCs (moDCs) that this potentiation is controlled exclusively via the p38/mitogen-activated protein kinase (MAPK) pathway. We identified MAPK kinase kinase 8 (MAP3K8) as a target gene of hypoxia-induced factor (HIF), a transcription factor controlled by oxygen tension, upstream of the p38/MAPK pathway. Hypoxia increased expression of MAP3K8 concomitant with the potentiation of TNF-α secretion. This potentiation was no longer observed upon siRNA silencing of MAP3K8 or with a small molecule inhibitor of this kinase, and this also decreased p38/MAPK phosphorylation. However, expression of DC maturation markers CD83, CD86, and HLA-DR were not changed by hypoxia. Since DCs play an important role in controlling T-cell activation and differentiation, our results provide novel insight in understanding T-cell responses in inflammation, cancer, autoimmune disease and other diseases where hypoxia is involved.

## Introduction

Dendritic cells (DCs) are the gatekeepers of the adaptive immune system, residing throughout peripheral tissues where they constantly sample for antigens [1,2]. When a DC encounters a foreign antigen, it secretes many inflammatory cytokines, including tumor necrosis factor α (TNF-α), that activate or repress the functions of other immune cells and contribute to an inflammatory disease state. Extracellular antigens can be ingested by endocytosis or phagocytosis, processed in the endolysosomal system and presented on MHC classes I and II on the surface of DCs. During this process, DCs traverse from the site of inflammation to the nearest draining lymph node, where they activate naive CD4^+^ and CD8^+^ T cells [3]. For the sampling of antigen and the traveling to the lymph node, a DC can pass many different tissues that display a wide range of oxygen levels, from almost atmospheric oxygen levels in the lung (20%) to hypoxic (<1%) oxygen tensions in the thymus and spleen [4]. On top of this, many disease states are characterized by low local oxygen levels. For example, the central region of solid tumors is very hypoxic due to a lack of vascularization [5,6]. Moreover, sites of infection and inflammation are often hypoxic microenvironments, due to the combination of reduced perfusion following physical damage and the high metabolic load of the activated inflammatory cells caused by the respiratory burst [7,8]. Hypoxia has been linked to many auto-inflammatory diseases, including diabetes and atherosclerosis [9–11]. In cancer, hypoxia induces the neovascularization that can promote tumor growth and possibly cancer cell metastasis [6]. Hypoxia can also be a direct driver of metastasis by inducing single-cell dissemination [12].

Oxygen levels are known to modulate many cellular processes, including metabolism, cell survival, and various signaling pathways [13,14]. The most important class of oxygen-sensitive signaling proteins is hypoxia-induced factor (HIF). HIFs are basic helix–loop–helix heterodimeric transcription factors involving three main subunits: HIF-1α, HIF-2α, and HIF-1β [13–15]. All three subunits are constitutionally expressed, but under normoxic conditions proline residues on the α-subunits are modified by prolyl hydroxylases (PHDs) in a process that consumes oxygen. After this hydroxylation, the von Hippel–Lindau E3 ligase (VHL) can trigger ubiquitination of the α-subunit leading to subsequent proteasomal degradation [13]. Under hypoxic conditions, PHD does not efficiently hydroxylate the α-subunit due to lack of oxygen, allowing HIF-1α or HIF-2α to dimerize with HIF-1β. These HIF complexes will then promote transcription of genes carrying hypoxic response elements (HREs) in their promoter regions [16–18].

Oxygen levels regulate the mammalian immune response, as it was recently shown that mice negative for HIF-1α fail to clear invasive bacterial infections [18]. Oxygen levels also modulate DC differentiation and the activation triggered by maturation stimuli such as lipopolysaccharide (LPS), a bacterial ligand of Toll-like receptor 4 (TLR4) [9,18–35]. Specifically, hypoxic DCs were shown to have increased levels of HIF-1α, metabolism shifted to glycolysis [30,31,33], and altered expression of chemokines and chemokine receptors affecting DC migration [27,28,30,32,34]. The effect of hypoxia on the expression of DC-maturation markers (e.g., CD80, CD83, CD86, MHC-II) is controversial, with both reductions [19,21,25–27,29,32], increases [30,31], and no effect [28,35] being reported. Similarly, both increased [31] and reduced [27,32] efficiencies of T-cell activation by hypoxic DCs, and both stimulatory [19,31,32] and inhibitory [21,25,27] effects of hypoxia on TLR-induced cytokine release (e.g., interleukin (IL)-6, IL-10, IL-12, TNF-α) have been reported.

The controversial findings of hypoxia on DC function likely arise from the fact that HIF not only modulates TLR signaling [36], but TLR stimulation in turn also affects HIF signaling [20,22,37]. TLR stimulation can promote the stabilization of HIF as well as up-regulate its expression [20,22]. The group of target genes regulated by HIF differs between activation of HIF via TLR stimulation or hypoxia [22] and this cross-talk between HIF and TLR signaling likely allows for fine-tuning of the cellular immune responses against different types of infections and disease states. However, the molecular mechanisms of the interplay between TLR and hypoxic signaling are still largely unknown. In the present study, we describe a pathway by which hypoxia potentiates TNF-α secretion by human monocyte-derived DCs (moDCs). MoDCs are differentiated from blood-isolated monocytes by culturing with IL-4 and granulocyte-macrophage colony-stimulating factor (GM-CSF). Although their physiological role is unclear, moDCs are capable of migration to lymph nodes and of both MHC-I and II presentation [19,23–28,30,32–35]. We show an increase in LPS-induced secretion of TNF-α by moDCs upon hypoxia, in line with previous findings [31,32]. Our data show that this potentiation is caused by hypoxia-promoted expression of mitogen-activated protein kinase (MAPK) kinase kinase 8 (MAP3K8; TPL-2; COT), which is a kinase involved in the MAP3K signaling pathway downstream of TLR4. Given the essential roles of DCs in T-cell activation and differentiation, our results are important for understanding T-cell responses in cancer, infection, autoimmunity, and other diseases and disorders associated with hypoxia.

## Experimental

### Cells and culture conditions

MoDCs were derived from peripheral blood mononuclear cells (PBMCs) obtained from buffy coats of healthy individuals as described previously [38]. Approval to conduct experiments with human blood samples was obtained from the blood bank and all experiments were conducted according to national and institutional guidelines. Informed consent was obtained from all blood donors by the Dutch blood bank. Samples were anonymized and none of the investigators could ascertain the identity of the blood donors. Briefly, peripheral blood leukocytes (PBLs) were separated from monocytes by a 1-h adhesion step at 5% CO_2_ and 37°C in RPMI-1640 (Thermo Fisher Scientific, Waltham, U.S.A.) with 2% human serum. Monocytes were differentiated into DCs by culturing for 6 days at 5% CO_2_ and 37°C in RPMI-1640 (Thermo Fisher Scientific) with 300 U/ml IL-4, 450 U/ml GM-CSF, 10% FBS (Greiner Bio-one, Kremsmünster, Austria), 2 mM UltraGlutamine (Lonza, Basel, Switzerland), and 1% antibiotic-antimycotic (Gibco by Life Technologies, Kremsmünster, Austria). For hypoxic culture, moDCs were kept overnight at 1% O_2_, 5% CO_2_, and 94% N_2_ at 37°C (CB53 incubator, Binder, Tuttlingen, Germany) in complete RPMI-1640 (Thermo Fisher Scientific) with 10% FBS (Greiner Bio-one), 2 mM UltraGlutamine (Lonza, Basel, Switzerland), and 1% antibiotic-antimycotic (Gibco by Life Technologies). For normoxic culture, cells were kept at atmospheric O_2_ (20%) with 5% CO_2_. To activate the DCs, 1 µg/ml of LPS (LPSs from *Escherichia coli* 0111:B4, Sigma–Aldrich, St. Louis, U.S.A.) was added to the medium. To quantitate cytokine production, cell culture supernatants were collected and stored at −80°C until analysis with IL-6 and TNF-α-specific ELISA (eBioscience, San Diego, U.S.A.).

### Inhibitors and antibodies

To inhibit p38 signaling, we used 1 µM SB203580 (Cell Signaling Technology, Danvers, U.S.A.). To inhibit IκB kinase (IKK), we used 100 nM IKK-16 (R&D Systems, Minneapolis, U.S.A.), to inhibit MAP3K8 we used 1 µM of Tpl2 kinase inhibitor (Cayman Chemical, Ann Arbor, U.S.A.), to inhibit Erk1/2 we used 250 nM of CAY10561 (Cayman Chemical). Primary antibodies used were: mouse-IgG1 anti-MAP3K8 (sc-373677, Santa Cruz Biotechnology, Dallas, U.S.A.), rabbit monoclonal IgG anti-p38/ MAPK (8690, Cell Signaling Technology), rabbit monoclonal IgG anti-p-p38/MAPK (Thr^180^/Tyr^182^) (4511, Cell Signaling Technology), rabbit polyclonal anti-p44/p42 MAPK (Erk1/2) (Cell Signaling Technology), mouse-IgG1 anti-p-p44/p42 MAPK (Erk1/2) (Thr^202^/Tyr^204^) (Cell Signaling Technology), rabbit monoclonal IgG anti-GAPDH (2118, Cell Signaling Technology). The following secondary antibodies were used: goat anti-rabbit or anti-mouse IgG (H+L) IRDye 800 CW (926-32211, 926-32210, LI-COR Biosciences, Lincoln, U.S.A.). Cell death was assessed with Zombie Violet fixable viability dye (BioLegend, San Diego, U.S.A.). The following directly labeled antibodies were used: CD11c-FITC (BD Biosciences, Franklin Lakes, U.S.A.), CD14-PE-Cy7 (Miltenyi Biotec, Bergisch Gladbach, Germany), CD16-APC (Miltenyi Biotec), CD11b-PE-Cy7 (Beckman Coulter, Brea, U.S.A.), CD68-APC (BioLegend), HLA-DR-PE (BD Biosciences), CD83-FITC (BD Biosciences), CD86-APC (BD Biosciences), TLR2-FITC (BioLegend), TLR4-APC (BioLegend).

### Western blot

SDS/PAGE with Western blot was used to analyze MAP3K8 knockdown efficiency and phosphorylation of p38 and Erk1/2 using 10% acrylamide gels. Proteins were then transferred to PVDF membranes (Immobilon-FL 0.45 µm, Merck, Burlington, U.S.A.), blocked for 1 h with 5% BSA in Tris/HCl-buffered saline and incubated overnight with primary antibodies (1:200 to 1:500 dilution). These were labeled by incubating with IRDye800–conjugated secondary antibodies (1:5000 dilution; LI-COR Biosciences) and analyzed using the Odyssey CLx Infrared Imaging System and ImageStudio Lite 5.0 analysis software (LI-COR Biosciences).

### Flow cytometry

For flow cytometry assays, moDCs were seeded at 100,000 cells per well in 100 µl of complete RPMI in a V-bottom 96-well plate. After incubation at hypoxic or atmospheric oxygen levels and with or without inhibitors, as described above, cells were centrifuged at 1500 rpm for 2 min at 4°C. Supernatant was discarded and cells were incubated for 30 min in the dark with PBS containing Zombie Violet fixable viability stain (BioLegend, 1:2000 dilution), then fixed with 4% PFA for 2 min and incubated for 10 min in the dark on ice with 50 µl of phosphate-buffered azide (PBA; PBS containing 0.5% BSA and 0.01% NaN_3_) containing 1% human serum to block Fc receptors. Then, cells were incubated for 30 min in the dark on ice with 50 µl of PBA containing directly labeled antibodies. To perform compensation and set gates, AbC Total Antibody Compensation beads (Thermo Fischer Scientific) were stained in the same way as the cell samples. Cells were washed with 100 µl PBA, resuspended in 60 µl PBA, and analyzed on a FACSCalibur or FACSVerse flow cytometer (BD Biosciences).

### Transfections

DCs were electroporated with siRNA against MAP3K8 (a 1:1:1 mix of GGCGUGUAAACUGAUCCCAGUAGAU, GGAAGGAGCUGGAACUUCCUGAGAA, and UGGUUGUCAUCAGUCAGAUAUGGAA; Thermo Fischer Scientific) using a Neon electroporation system (Thermo Fischer Scientific). Briefly, DCs were thawed from liquid nitrogen storage, washed once with PBS and resuspended in R buffer (from the Neon kit) at 0.5–1.0 × 10^6^ cells in 120 µl volume. Four micrograms of plasmid DNA or 20 pmol siRNA mix was added and the cells were electroporated with two pulses of 1000 V at 40 ms pulse width. Cells were then transferred to pre-warmed RPMI-1640 containing 2 mM UltraGlutamine. Four hours post transfection, an equal amount of complete RPMI-1640 was added to the cells. Experiments were performed 24 h post-transfection.

### Primers and PCRs

MAP3K8 expression in untreated moDCs was checked with PCR on cDNA using primers for MAP3K8 (CTCCCCAAAATGGACGTTACC and GGATTTCCACATCAGATGGCTTA). Expression levels were quantitated with real-time quantitative PCR (RT-qPCR) using validated primers for MAP3K8 (GAGCGTTCTAAGTCTCTGCTG and GCAAGCAAATCCTCCACAGTTC), TBP (GAGCCAAGAGTGAAGAACAGTC and GCTCCCCACCATATTCTGAATCT) and GUSB (GACACGCTAGAGCATGAGGG and GGGTGAGTGTGTTGTTGATGG). Briefly, mRNA was isolated from DCs using a Quick-RNA MiniPrep Plus kit (Zymo Research, Irvine, U.S.A.) and cDNA was generated in a standard reverse transcriptase reaction. qPCRs were done on a CFX96 Real-Time PCR Detection System (Bio-Rad, Hercules, U.S.A.), using FastStart SYBR Green Master mix (Hoffmann-La Roche, Basel, Switzerland) and analyzed in CFX Manager software version 3.1 (Bio-Rad, Hercules, U.S.A.). Gene expression was first normalized (ΔΔCq) to housekeeping genes (*TBP* and *GUSB*) validated with geNorm analysis, followed by normalization to untreated samples.

### Statistical analysis

For datasets with two paired conditions, paired two-sided Student’s *t* tests were applied to assess significance when data followed a normal distribution, otherwise Wilcoxon’s matched pairs signed-rank tests were used. One-way ANOVA with Bonferroni’s post-hoc tests were applied for multiple comparisons. Box plots indicate mean with whiskers depicting minimal and maximal values. A value of *P*<0.05 was considered statistically significant (*P<0.05, **P<0.01, ***P<0.001).

## Results

### TLR4-triggered TNF-α secretion is potentiated by hypoxia

**Figure 1:**
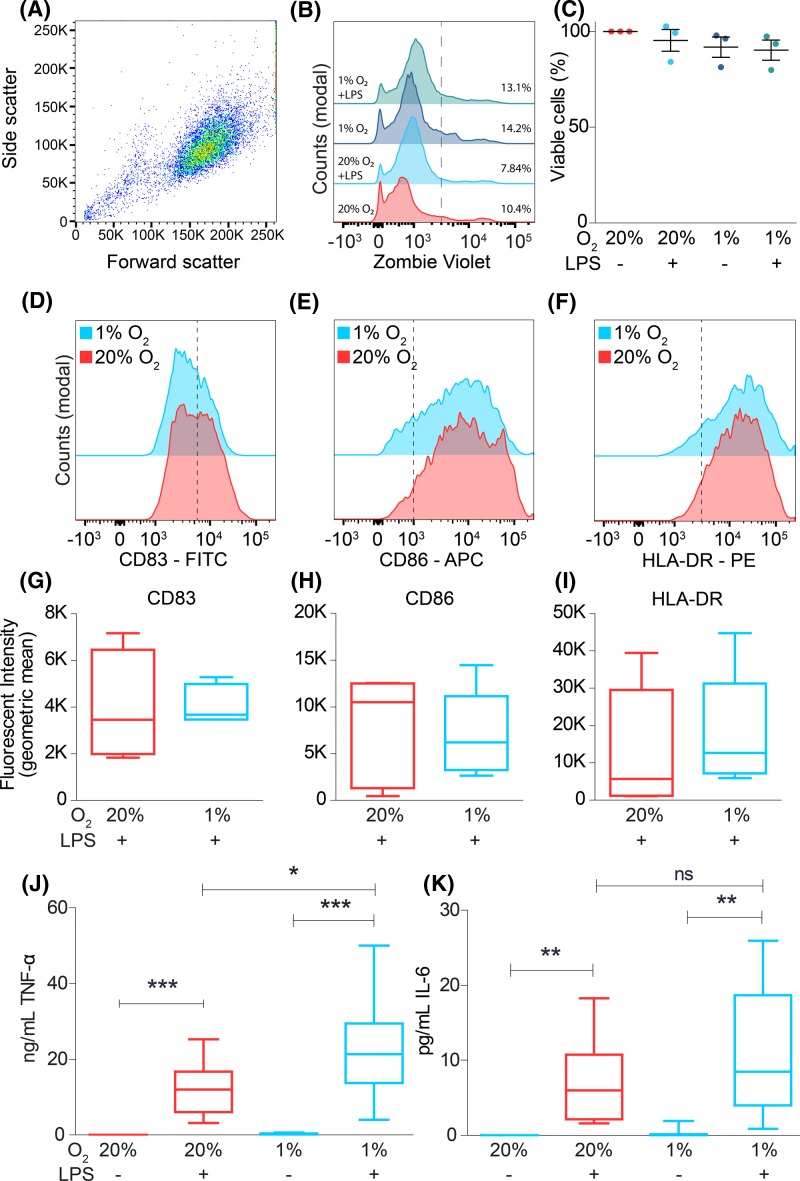
Hypoxia potentiates LPS-induced TNF-α secretion (**A**–**C**) Cell viability of moDCs during atmospheric (20% O_2_) and hypoxic (1% O_2_) oxygen levels in presence or absence of LPS, determined by Zombie Violet as analyzed by flow cytometry. Representative dot plot (A), histograms (B), and quantitation for three donors ((C); average ± S.E.M.) are shown. (**D**–**I**) LPS-induced maturation of moDCs cultured at atmospheric or hypoxic oxygen levels, determined by expression levels of maturation markers CD83 (D,G), CD86 (E,H), and HLA-DR (F,I). Representative histograms showed quantitation for five donors. (**J**,**K**) Secretion by ELISA of the pro-inflammatory cytokines TNF-α (J) and IL-6 (K) from moDCs cultured at atmospheric or hypoxic oxygen levels. Quantitation from ten donors (average ± S.E.M.). *P<0.05; **P<0.01; ***P<0.001. Abbreviation: ns, not significant.

We started by studying the interplay of hypoxia and infection on the secretion of IL-6 and TNF-α. We used DCs differentiated from monocytes isolated from the blood of healthy volunteers by culturing in the presence of IL-4 and GM-CSF (moDCs). MoDCs are capable of endocytosis, phagocytosis, antigen (cross-)presentation and activation of T cells, and are a widely used model system for studying hypoxia on DC function [19,23–28,30,32–35]. Although moDCs are also found *in vivo*, they differ from conventional blood-circulating DC subsets and there has been an ongoing debate about their exact physiological role [39–41]. We characterized our moDC cultures by measuring expression of several DC, monocyte and macrophage surface markers. MoDCs were found to be a homogeneous population of CD11c^+^/CD11b^+^/CD16^−^/CD68^−^ cells, of which ∼87% also expressed CD14^+^ (Supplementary Figure S1A–I). These results show that our cells are composed mainly of DC-like cells which express high levels of the DC marker CD11c. Monocytic lineage markers CD14 and CD11b were also highly expressed, whereas only low expression of the monocyte/macrophage markers CD16 and CD68 was observed. The moDCs were cultured in a hypoxic environment of 1% oxygen, or in an atmospheric environment of 20% oxygen. Similar to previous reports [19,25,28,31,35], flow cytometry experiments showed that culturing DCs overnight under hypoxic conditions did not significantly affect cell viability (Figure 1A–C). Culturing under hypoxic conditions trended toward decreased CD14 expression and a non-consistent increase in CD68 expression, however these differences are not significant. Expression of all other tested surface markers was unaltered (Supplementary Figure S1J–S).

To stimulate an inflammatory response, we cultured the moDCs in the presence of LPS. Compared with LPS at atmospheric oxygen levels, LPS with hypoxia did not result in significant changes in surface expression of the maturation markers CD83 and CD86 (Figure 1D,E,G,H), as reported previously [26,28,30], but contrasting with other studies [19,21,25,31,32]. Surface levels of MHC-II were also not changed by hypoxia (Figure 1F,I), which again corresponds with some studies [28,30], while other studies report higher [31] or lower [21,25–27,29,32] MHC-II levels upon hypoxia. We observed the strongest potentiation for production of TNF-α, where hypoxia resulted in an approximately two-fold significant increase in LPS-triggered TNF-α secretion compared with atmospheric oxygen (Figure 1J). The increase in LPS-induced IL-6 secretion upon hypoxia was not significant compared with atmospheric oxygen levels (Figure 1K). A potentiation in TLR-stimulated TNF-α secretion by DCs upon hypoxia has been reported previously in some [31,32], but not all [21,27], studies. Moreover, hypoxia-mediated potentiation of TNF-α secretion has been observed for macrophages [17], osteoblasts [42], and human hepatocellular carcinoma cells [36].

### SB203580, an inhibitor for p38/MAPK, blocks hypoxia-potentiated TNF-α secretion

**Figure 2:**
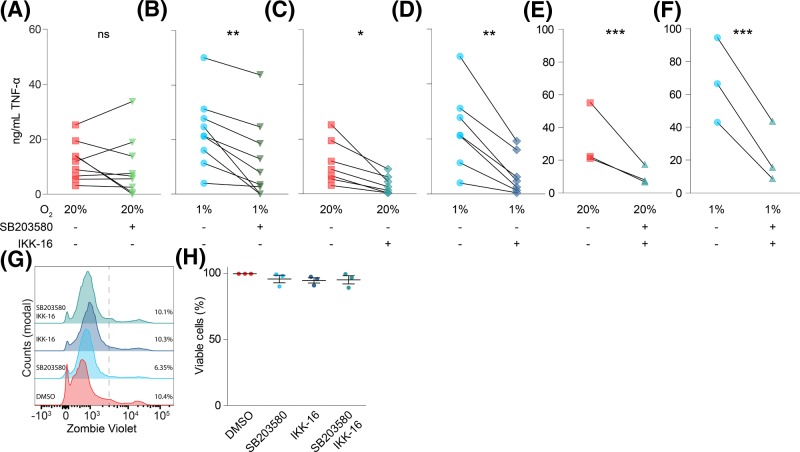
Hypoxia-mediated potentiation of TNF-α secretion is controlled by p38/MAPK (**A**–**F**) Effects of inhibitors of p38/MAPK (1 µM SB203580; (A,B,E,F)) and/or IKK (100 nM IKK-16; (C–F)) on LPS-induced TNF-α secretion by moDCs cultured under atmospheric (20% O_2_; (A,C,E)) or hypoxic (1% O_2_; (B,D,F)) oxygen levels. Individual donors shown. (**G**,**H**) Effects of SB203580 and/or IKK-16 on moDC viability, determined by flow cytometry labeling with Zombie Violet as analyzed by flow cytometry. Representative histograms (G) and quantitation for four donors ((H); average ± S.E.M.). *P<0.05; **P<0.01; ***P<0.001. Abbreviaton: ns, not significant.

TLR4 stimulation triggers TNF-α secretion via two distinct signaling pathways. In the first pathway, TNF-α expression is up-regulated by activated nuclear factor κB (NF-κB) following IKK phosphorylation by TLR4. In the second pathway, TNF-α secretion is mediated via p38/MAPK signaling, which is activated in tandem with NF-κB following TLR4 stimulation [43,44]. We used specific inhibitors to elucidate the contribution of each pathway in the hypoxia-mediated potentiation of TNF-α secretion. Inhibition of p38/MAPK with SB203580, which inhibits the downstream catalytic activity of p38 [45,46], did not affect LPS-triggered TNF-α secretion under atmospheric oxygen levels (Figure 2A). In contrast, under hypoxic conditions, SB203580 resulted in a consistent and significant reduction in TNF-α secretion (Figure 2B). Blockage of IKK, which is responsible for activation of NF-κB [47,48], by IKK-16 blocked TNF-α secretion almost completely (to ∼20% of untreated) both under atmospheric and hypoxic conditions (Figure 2C,D). Simultaneous addition of both SB203580 and IKK-16 had a comparable effect (Figure 2E,F). The residual TNF-α secretion might be due to incomplete inhibition of the NF-κB and/or p38/MAPK pathways or due to the involvement of other signaling pathways. Under our conditions, SB203580 and IKK-16 did not affect the viability of the cells (Figure 2G,H). These findings suggest that the hypoxia-mediated potentiation of TNF-α release primarily occurs via increased activation of the p38/MAPK signaling pathway, while the IKK signaling pathway is activated by LPS irrespective of oxygen levels. In-line with this, hypoxia was reported to promote TLR2 and TLR4-induced pro-inflammatory activation of macrophages via the p38/MAPK pathway, but not via IKK signaling [49]. In contrast with macrophages [50], surface expression of TLR2 and TLR4 was unaffected by hypoxia (Supplementary Figure S2A–D), suggesting that the increased TNF-α secretion was not caused by increased expression of these pattern recognition receptors.

**Figure 3:**
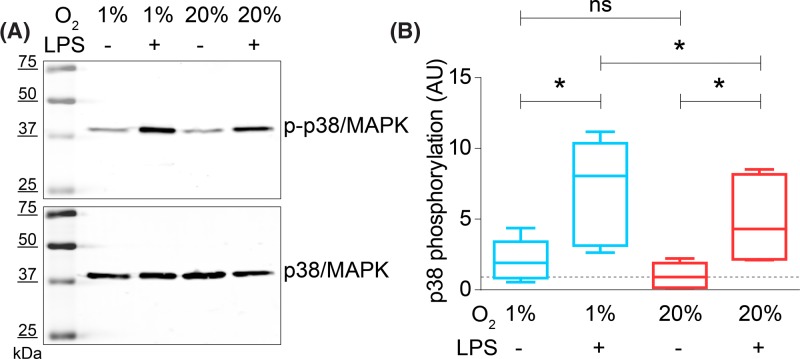
LPS-induced phosphorylation of p38/MAPK is increased at hypoxia (**A**) Representative Western blot of moDCs cultured at atmospheric (20% O_2_) or hypoxic (1% O_2_) oxygen levels and in presence or absence of LPS. The blot was stained with an antibody specific for p-p38/MAPK (top) and total p38/MAPK (bottom). (**B**) Quantitation of (A) for five donors. p38/MAPK phosphorylation during hypoxic inflammation, normalized to unstimulated cells at atmospheric oxygen levels (LPS-, 20% O_2_). *P<0.05; **P<0.01; ***P<0.001. Abbreviation: ns, not significant.

To directly investigate the activation of the p38/MAPK pathway by hypoxia and LPS, we determined activation of p38 by Western blot with phosphospecific antibodies (Figure 3A). As described previously, LPS treatment of DCs leads to activation of p38 by phosphorylation of the residues Thr^180^ and Tyr^182^ [51]. In-line with this, we observed a three- to four-fold increase in p-p38 upon LPS stimulation compared with unstimulated cells at atmospheric oxygen levels. Culturing the cells under hypoxic conditions in presence of LPS led to a further significant increase in p38 phosphorylation by ∼50%, whereas this increase was non-significant in absence of LPS (Figure 3B).

### MAP3K8 is an HIF target gene and is overexpressed at hypoxic inflammation

**Figure 4:**
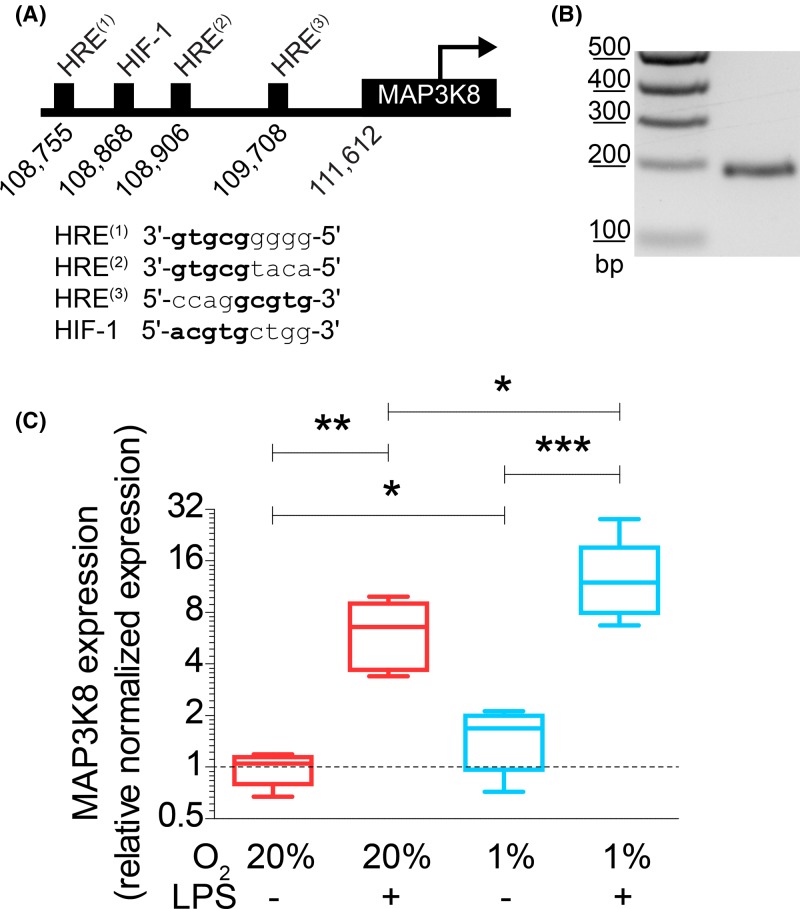
Hypoxia increases expression of HIF target gene *MAP3K8* (**A**) Positions and sequences of HRE and HIF-binding site (HIF-1) in the promoter region of human MAP3K8. (**B**) *MAP3K8* mRNA expression by moDCs as determined by PCR on cDNA obtained from unstimulated moDCs. Expected band size is 201 bp. (**C**) mRNA expression levels of MAP3K8 in moDCs cultured at atmospheric or hypoxic conditions in absence or presence of LPS determined by RT-qPCR. Quantitation from six donors. *P<0.05, **P<0.01, ***P<0.001.

Hypoxia is well-known to increase expression of genes with an HRE in their promoter via the transcription factor HIF [15,16]. We therefore searched for genes coding for components in the p38/MAPK pathway carrying one or more HREs in their promoter. We identified MAP3K8 as a candidate gene downstream of HIF, since it is known to have a HIF-1β binding site and is up-regulated under hypoxia [17,52,53]. Indeed, we identified three HREs and an HIF-1-specific binding site upstream of the *MAP3K8* gene on chromosome 10 (GenBank accession AL161651.13) using PROMO (Figure 4A) [54,55]. Reverse transcriptase (RT-) PCR showed that MAP3K8 is expressed in moDCs (Figure 4B).

Since MAP3K8 is a key mediator of p38/MAPK signaling, we hypothesized that the hypoxia-induced TNF-α potentiation occurred via increased expression of MAP3K8. To address this hypothesis, we cultured moDCs under atmospheric or hypoxic conditions and with or without addition of LPS, followed by mRNA isolation to analyze expression of MAP3K8 by RT-qPCR. Hypoxia led to a significant approximately two-fold increased expression of MAP3K8 in absence of LPS (Figure 4C). In the presence of LPS, this increase was even more pronounced and we observed an average ∼14-fold increase in MAP3K8 expression. Overall, the expression of MAP3K8 mirrors the potentiation of TNF-α secretion by a combination of LPS and hypoxia (Figure 1J) and is in qualitative agreement with the increased phosphorylation of p38 (Figure 3B). These findings support our hypothesis that MAP3K8 potentiates LPS-induced TNF-α secretion under hypoxic conditions.

### MAP3K8 directly links HIF signaling to the p38/MAPK pathway

**Figure 5:**
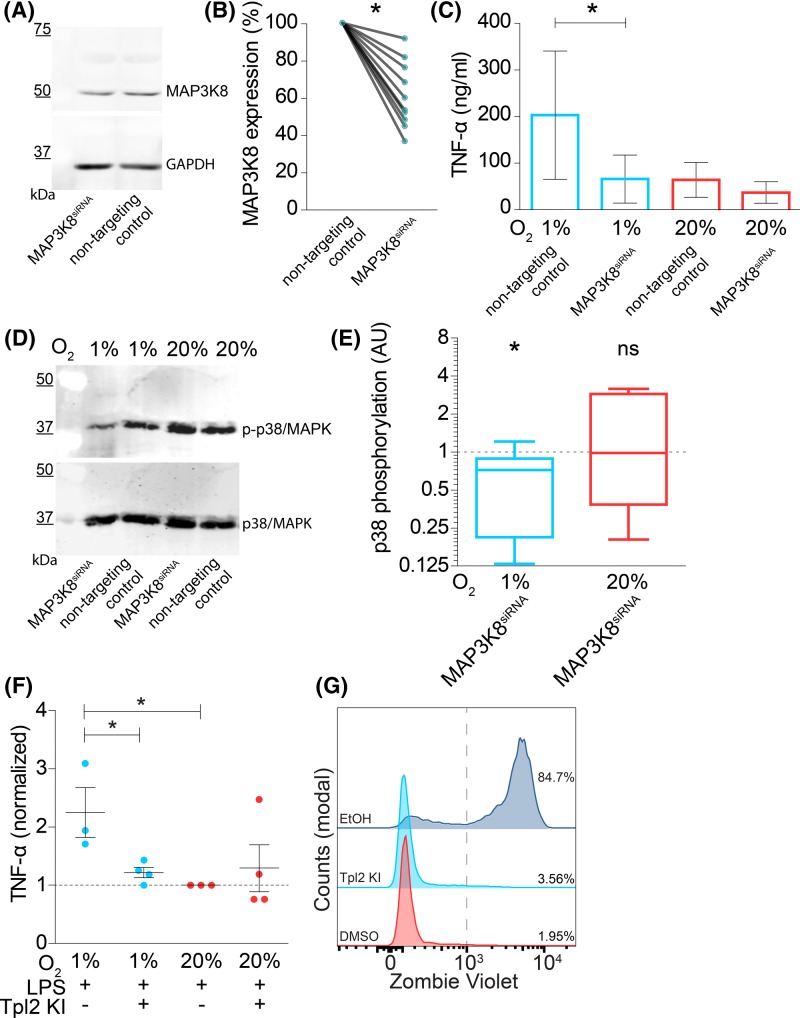
MAP3K8 knockdown in moDCs blocks hypoxia-induced potentiation of LPS-induced TNF-α secretion (**A**) Representative Western blot of siRNA knockdown of MAP3K8 in moDCs (MAP3K8^siRNA^) stained with an antibody specific for MAP3K8 (top) and loading control (GAPDH; bottom). MoDCs were transfected with non-targetting siRNA as negative control. (**B**) Quantitation of (A) by band intensities normalized to GAPDH and shown relative to non-targetting control. Individual donors are shown. Compared using Wilcoxon’s matched-pairs signed rank test. (**C**) TNF-α secretion by moDCs with or without MAP3K8^siRNA^ and cultured at atmospheric (20% O_2_) or hypoxic (1% O_2_) oxygen levels. Quantitation of 14 donors (average ± S.E.M.). (**D**) Phosphorylation of p38/MAPK in moDCs with or without MAP3K8^siRNA^ and cultured at atmospheric (20% O_2_) or hypoxic (1% O_2_) oxygen levels. Representative Western blot probed for phosphorylated and total p38/MAPK shown. (**E**) Quantitation of (D) for six donors. Shown are the ratios of p38/MAPK phosphorylation for MAP3K8^siRNA^ moDCs compared with non-targetting siRNA controls. (**F**) TNF-α secretion by moDCs following overnight incubation with LPS and Tpl2 kinase inhibitor (Tpl2 KI) normalized to atmospheric oxygen condition (average ± S.E.M.). (**G**) Representative flow cytometry histogram showing Zombie Violet cell viability staining on moDCs incubated overnight with Tpl2 KI, DMSO negative control, or 2 min 70% ethanol (EtOH) as positive control. *P<0.05. Abbreviation: ns, not significant.

To obtain direct evidence that MAP3K8 is a key factor linking hypoxia to potentiation of LPS-stimulated TNF-α secretion, we performed knockdown experiments in moDCs where we targetted MAP3K8 with siRNA (MAP3K8^siRNA^). As a negative control, we transfected the moDCs with non-targetting siRNA. With this technique, we obtained an average reduction in ∼40% of MAP3K8 at the protein level, as quantitated by Western blot (Figure 5A,B). MAP3K8^siRNA^ blocked the hypoxia-mediated potentiation of LPS-stimulated TNF-α secretion (Figure 5C). Phosphorylation of p38 was also reduced in MAP3K8^siRNA^ moDCs following LPS stimulation at hypoxic, but not at atmospheric oxygen levels (Figure 5D,E).

We further validated the role of MAP3K8 in potentiation of TNF-α secretion with the small molecule inhibitor 4-[(3-chloro-4-fluorophenyl)amino]-6-[(3-pyridinylmethyl)amino]-1,7-naphthyridine-3-carbonitrile (Tpl2 kinase inhibitor). This molecule inhibits MAP3K8 by blocking binding of ATP to MAP3K8 [56] and did not affect cell viability (Figure 5G). In-line with the results obtained with siRNA knockdown, we observed that overnight incubation with this inhibitor together with LPS stimulation decreased the hypoxia-mediated potentiation of TNF-α secretion (Figure 5F). We also investigated phosphorylation of p38/MAPK following incubation with this inhibitor (Supplementary Figure S3A,B), and while we observed a reduction in LPS-mediated p38/MAPK phosphorylation under hypoxic conditions, this was not statistically significant. Together, these findings support that MAP3K8 promotes TLR-mediated TNF-α secretion under hypoxic conditions.

Another major downstream target of MAP3K8 is the MEK/Erk pathway, which is described in literature to promote neutrophil development and LPS-mediated TNF-α secretion [57–59]. Therefore, we also probed for Erk1/2 phosphorylation in moDCs stimulated with LPS and cultured at hypoxic and atmospheric oxygen levels (Supplementary Figure S3C,D). We used inhibitors for Erk1/2 (CAY10561) [60] and MAP3K8 (Tpl2 kinase inhibitor) in this experiment and performed a TNF-α ELISA. MoDC viability was unaffected by these small molecule inhibitors (Supplementary Figure S3 and Figure 5G). However, we observed only low and inconsistent activation of Erk1/2 following LPS and this was not altered by hypoxic culture. In contrast, a positive control of starved HeLa cells exposed to serum showed clear Erk1/2 phosphorylation. Moreover, the increased TNF-α secretion under hypoxic conditions was not modified by inhibition of Erk1/2 (Supplementary Figure S3E). Phosphorylation of p38/MAPK was also unaffected by this inhibitor (Supplementary Figure S3A,B). These results show that LPS-mediated activation of the p38 pathway is specifically potentiated via MAP3K8 under hypoxic conditions.

## Discussion

Hypoxia is capable of both potentiating and suppressing the response of immune cells and other cells to TLR stimuli. In primary human macrophages, the TLR2- and 4-induced expression and secretion of various pro-inflammatory cytokines (amongst others IL-6, IL-8, and IL-1β) was shown to be increased by hypoxia. In osteoclasts, hypoxia induced TNF-α expression and this depended on HIF-1α expression [42]. However, the effects of hypoxia on human DC function are more controversial, and both the inhibition [21,25,27,32] as well as stimulation [19,31,32] of TLR-induced cytokine secretion by hypoxia have been described (e.g., for IL-6, IL-10, IL-12, TNF-α). Antigen uptake by DCs was shown to be less efficient in hypoxic DCs [27,30], although Bosseto et al. [19] showed that uptake was not affected for *Leishmania* infections. The effects of hypoxia on the subsequent activation of T cells by DCs are equally controversial, and Jantsch et al. [31] described an increase in activation, while others reported a decrease [27,32]. The relatively modest effects observed in this study may explain the many contradictory reports on the effects of hypoxia on immune cells.

The divergent effects of hypoxia on immune cell regulation are probably caused by the complex interplay of hypoxia on intracellular signaling. In addition to the stabilization of HIF transcription factors, hypoxia causes a metabolic shift toward anaerobic glycolysis, as also shown for DCs [61]. This metabolic shift can further affect the secretion of cytokines, as shown for IL-1β secretion by macrophages [62]. Moreover, for immune phagocytes such as DCs, TLR signaling can cause local hypoxia due to the large oxygen consumption by NAPDH oxidases that produce reactive oxygen species (ROS) during the respiratory burst [20,24]. These ROS in turn can act as second messengers, further modulating the transcriptional profile of immune cells, and for instance the hypoxia-induced activation of NFκB is redox sensitive [63]. TLR-signaling can also directly affect HIF signaling via post-translational regulation and increased expression of HIF [22,64–66]. Additionally, hypoxia was shown to up-regulate both HIF and TLR4 expression in macrophages [50] and synergistic induction of HIF-1α activity by hypoxia and TLR4 stimulation has been shown in macrophages [67]. Finally, TNF-α can already induce HIF-1α expression under atmospheric conditions [68]. All these interacting pathways make it difficult to discern cellular responses to hypoxia from those caused by TLR signaling.

**Figure 6:**
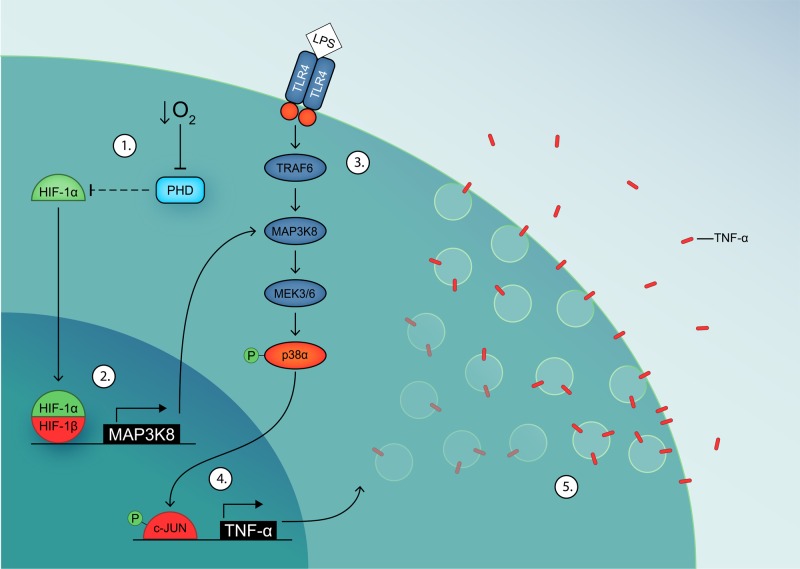
Model of MAP3K8-mediated potentiation of TNF-α secretion in hypoxic inflammation (1) Hypoxia inhibits PHD activity, leading to stabilization of HIF-1α. (2) HIF-1α dimerizes with HIF-1β and up-regulates MAP3K8 expression, which potentiates the p38/MAPK signaling cascade. (3) LPS-induced dimerization of TLR4 activates the p38/MAPK signaling cascade. (4) c-JUN is phosphorylated by p-p38α and up-regulates TNF-α expression. (5) TNF-α is transported to the plasma membrane and released.

Because of the multifactorial responses of cells to hypoxia, the molecular mechanisms of hypoxia-mediated regulation of immune cell differentiation are still largely unknown. In corneal epithelial cells, the hypoxia-mediated attenuation of NF-κB and Erk1/2 activation and of IL-6 and IL-8 secretion are dependent on myeloid differentiation primary response gene 88 (*MyD88*) downstream of TLR4. In PC12 cells and primary human macrophages, the potentiation of inflammatory cytokine secretion (IL-1β, IL-6, IL-8) by hypoxia is controlled specifically via p38/MAPK and c-Jun N-terminal kinase (JNK) signaling, but not via IκBα [49,69]. In this study, we identified a new molecular link between hypoxia and TLR signaling in human moDCs (Figure 6). We found that MAP3K8 expression is directly regulated by hypoxia via several HREs, resulting in increased expression of MAP3K8 [17]. As the MAP3K8 kinase is involved in activation of the p38/MAPK pathway [70,71], its up-regulation results in increased transcription of pro-inflammatory cytokines [57]. In this way, hypoxia-induced MAP3K8 up-regulation sensitizes the cells to TLR stimulation. Since studies with MAP3K8 knockout mice demonstrated that long-term secretion of TNF-α, but not IL-6, is regulated by MAP3K8 [72], our findings explain why hypoxia leads to a potentiation of TLR-stimulated TNF-α secretion, but not or less of IL-6 secretion.

Cancer cells express p38/MAPK, and this pathway is activated by hypoxia [73,74]. MAPK signaling can induce angiogenesis and metastasis, as well as tolerance by polarization of macrophages to the tolerogenic M2 subtype and of T cells to Th17 cells [12,75]. In this way, MAP3K8 expression promotes tumor growth and progression as well as immune suppression [76,77]. On the other hand, MAPK signaling can also have tumor suppressing functions: its expression is associated with lung cancer patient survival via a p38 signaling pathway downstream of JNK [78] and it suppresses colitis-associated tumorigenesis [79]. In-line with this, MAP3K8^−/−^ mice show more tumor initiation and faster progression of chemically induced skin cancer [80].

MAP3K8 is also involved in autoimmunity. For example, MAP3K8 links IL-17 receptor signaling to TAK1 activation, which leads to increased JNK, p38, and NFκB activity in Th17 cells, promoting autoimmune neuroinflammation [81,82]. Moreover, MAP3K8 activity in T cells limits regulatory T cell (Treg) function by inhibiting FoxP3 expression and lowering production of the immunosuppressive cytokines IL-10 and cytotoxic T lymphocyte-associated protein 4 (CTLA-4) [83]. In addition, TNF-α plays a pivotal role in a variety of autoimmune diseases, causing sustained inflammation and tissue damage [84]. In many autoimmune diseases, a negative feedback loop between tissue damage and inflammation exists, where MAP3K8 promotes myeloperoxidase activity, ROS production, and recruitment of neutrophils and macrophages, causing tissue damage [72]. This tissue damage and ROS production might subsequently lead to local hypoxia which further up-regulates MAP3K8 activity, contributing to the localized hypoxia observed in many autoimmune diseases. In-line with this, patients suffering from systemic sclerosis and cerebrovascular inflammation show clear signs of localized hypoxia, and fibroblasts from these patients show marked increases in activation of p38 [85–87]. In addition, MAP3K8^−/−^ mice have reduced amounts of circulating neutrophils, which display impaired sensitivity to chemokines and lower TNF-α secretion [57,58]. Bone-marrow derived DCs of MAP3K8^−/−^ mice also show impaired TNF-α secretion [88] and increased IL-12 [89]. It would be interesting to study DC function in these MAP3K8^−/−^ mice under hypoxic conditions to confirm our *in vitro* findings.

In conclusion, we show that hypoxia potentiates LPS-stimulated TNF-α secretion in human moDCs by up-regulating expression of MAP3K8, a serine/threonine kinase capable of activating the p38/MAPK signaling pathway. Since our data provide a mechanistic link between hypoxia and immune cell function, and since these are important factors in infection, cancer, and autoimmune disease, MAP3K8 might be a promising therapeutic target for treatment of these diseases.

## Supporting information

**Supplementary Figure:** 

**Supplementary Figure:** 

**Supplementary Figure:** 

## Abbreviations

DC: dendritic cell
GAPDH: glyceraldehyde 3-phosphate dehydrogenase
GM-CSF: granulocyte-macrophage colony-stimulating factor
HIF: hypoxia-induced factor
HRE: hypoxic response element
IL: interleukin
IKK: IκB kinase
JNK: c-Jun N-terminal kinase
LPS: lipopolysaccharide
MAPK: mitogen-activated protein kinase
MAP3K8: MAPK kinase kinase 8
moDC: monocyte-derived DC
NF-κB: nuclear factor κB
PBA: phosphate-buffered azide
PHD: prolyl hydroxylase
qPCR: quantitative polymerase chain reaction
ROS: reactive oxygen species
RT-qPCR: real-time quantitative PCR
TLR4: toll-like receptor 4
TNF-α: tumor necrosis factor α
