# Supplementary figures and images for: Hypoxia potentiates monocyte-derived dendritic cells for release of tumor necrosis factor α via MAP3K8
Source: Biosci Rep. 2018 Dec 14;38(6):BSR20182019. doi: 10.1042/BSR20182019 (PMC6294625; doi:10.1042/BSR20182019)

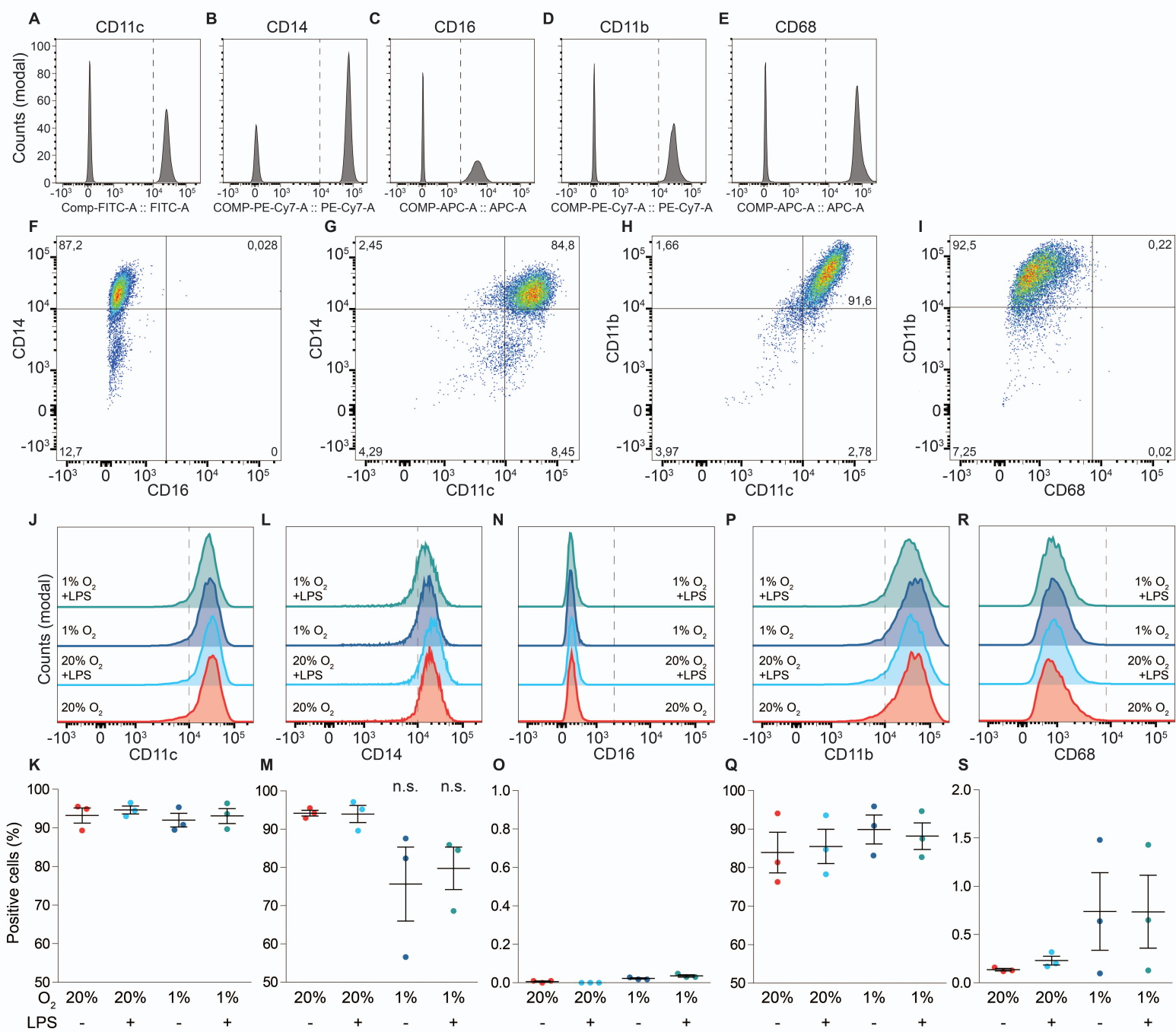

**A**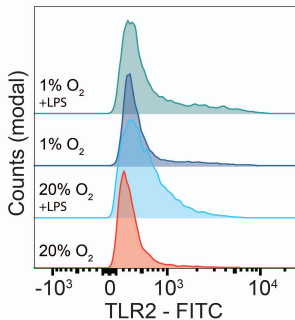**B**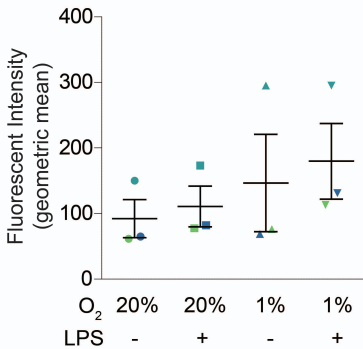**C**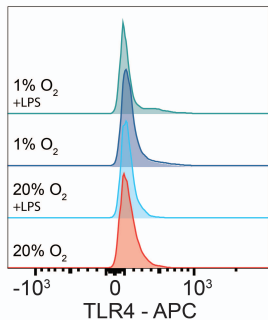**D**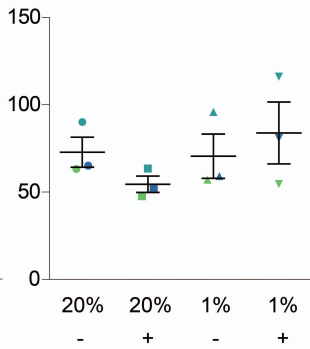

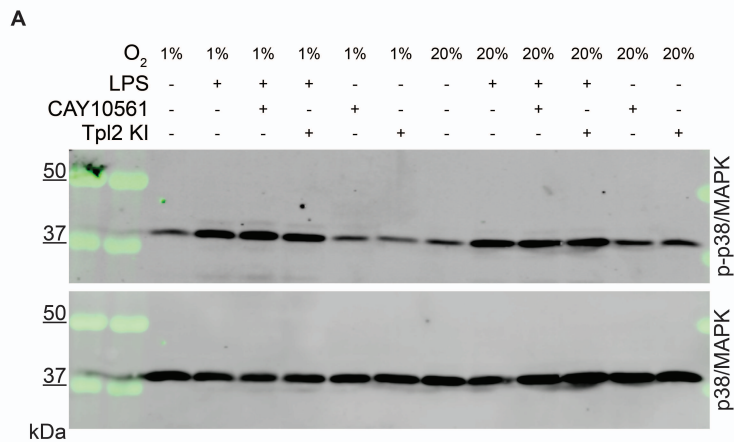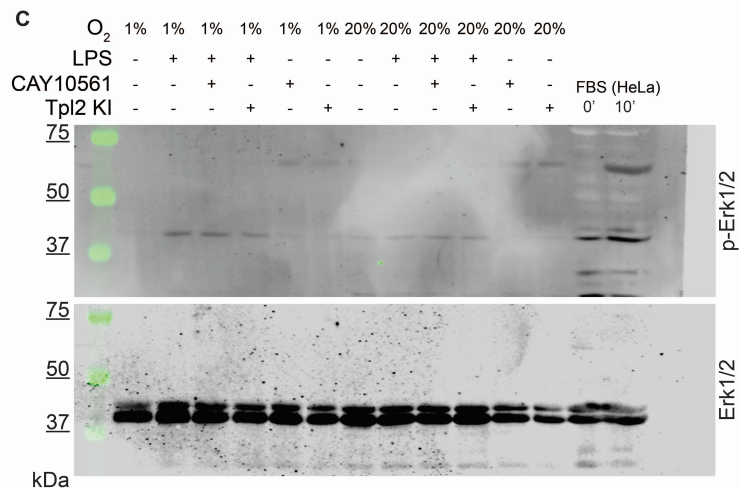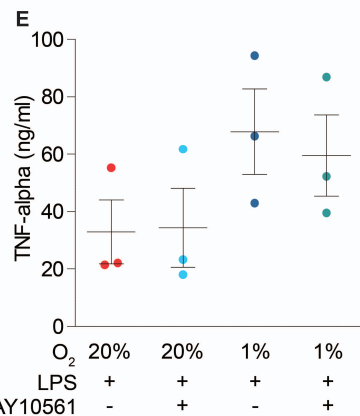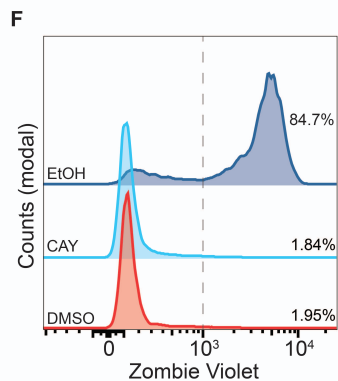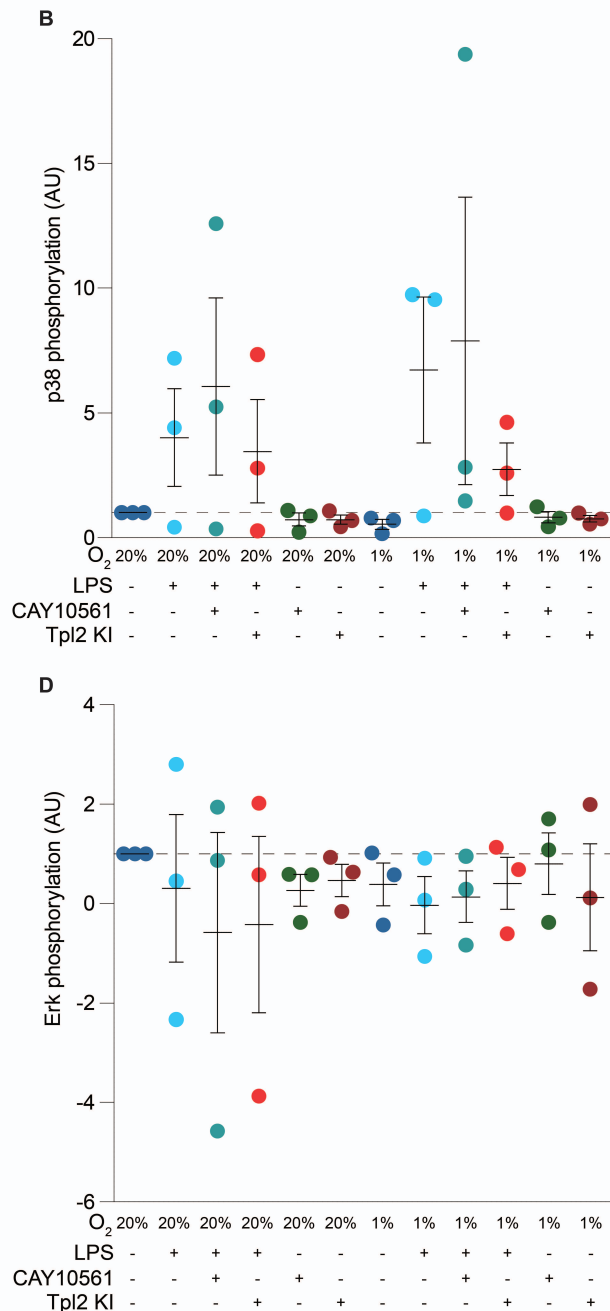

Supplement: Supplementary file 1 [file bsr20182019_Supp1.pdf]
